# Plant Heat Adaptation: priming in response to heat stress

**DOI:** 10.12688/f1000research.7526.1

**Published:** 2016-04-18

**Authors:** Isabel Bäurle

**Affiliations:** 1 Institute for Biochemistry and Biology, University of Potsdam, Potsdam, 14476, Germany

**Keywords:** Abiotic stress, heat stress, thermo-priming, heat stress priming, heat stress memory, plant heat adaptation

## Abstract

Abiotic stress is a major threat to crop yield stability. Plants can be primed by heat stress, which enables them to subsequently survive temperatures that are lethal to a plant in the naïve state. This is a rapid response that has been known for many years and that is highly conserved across kingdoms. Interestingly, recent studies in Arabidopsis and rice show that this thermo-priming lasts for several days at normal growth temperatures and that it is an active process that is genetically separable from the priming itself. This is referred to as maintenance of acquired thermotolerance or heat stress memory. Such a memory conceivably has adaptive advantages under natural conditions, where heat stress often is chronic or recurring. In this review, I will focus on recent advances in the mechanistic understanding of heat stress memory.

## Introduction

Plants are sessile organisms that gauge and adapt to stressful environmental conditions in order to ensure survival and reproductive success. Such stressful conditions include extreme temperatures, drought, salinity, and pathogen and herbivore attacks. In nature, these are often chronic or recurring. Thus, plants have evolved strategies to cope with recurring stress. One such strategy is priming, where a past stress exposure modifies responses to a later stress event ^1– 5^. The term priming was coined in the context of pathogen defense, where a transient assault primes a plant to respond more efficiently in response to a future pathogen attack ^6^. In the last few years, the term priming has been increasingly used to describe analogous phenomena that occur in response to other stresses ^5^. Priming involves a lag/memory phase that separates the priming event and the second stress event. Research into different priming phenomena and their respective molecular bases has recently received increasing attention ^7– 9^. However, the molecular mechanisms underlying plant stress priming and memory are still largely unknown. In this context, the term memory is defined operationally as a phenomenon where information is perceived, stored, and later retrieved, as shown by a modified response to a second stimulus ^10^. Mechanistically, stress memory may take place at different levels, ranging from metabolites and protein stability to chromatin complexes. In this review, recent progress in the field of priming by and memory of heat stress (HS) will be discussed. HS is a severe threat to global agriculture, and its significance will likely increase with climate change ^11^. HS is detrimental, especially in combination with the lack of a water supply ^12^ and during specific developmental stages such as pollen development ^11, 13, 14^. It is therefore of the utmost importance to increase our understanding of the molecular basis of plant responses to HS in order to develop strategies to improve stress resistance in crop plants ^15^.

## Heat stress priming and heat stress memory

Moderate HS primes a plant to subsequently withstand high temperatures that are lethal to an unadapted plant ^16^. This is also referred to as acquisition of thermotolerance. After returning to non-stress temperatures, the primed state is maintained over several days (referred to as maintenance of acquired thermotolerance or HS memory), and this maintenance is genetically separable from HS priming ^17– 19^. The responses to acute HS have been studied intensively over the last few decades and are covered in several recent reviews ^20– 22^. In brief, HS priming involves the activation of heat shock transcription factors (HSFs) that induce the expression of heat shock proteins (HSPs), which in turn assist protein homeostasis through their chaperone activities ^22, 23^. This HS response is conserved in plants, animals, and fungi ^20^. Whereas yeast and animals have only one or a few copies of HSF genes, plants typically contain more than 20 members of this protein family ^21^. In *Arabidopsis thaliana*, at least eight HSFs are involved in the responses to HS ^17, 24– 27^. HS priming is thought to be mediated primarily through HSFA1 isoforms ^27^.

Whereas the molecular events that lead to HS priming are relatively well understood, little is known about the mechanism of HS memory (i.e. the maintenance of the primed state after HS). *HSFA2* is the most strongly heat-induced HSF ^24, 28^. Interestingly, *HSFA2* is required not for HS priming but specifically for HS memory ^17^. Microarray analyses have identified a number of HS memory-related genes that were classified on the basis of their sustained induction after HS, which lasts for at least 3 days ^19^. They comprise many genes encoding small HSPs (such as HSP21, HSP22.0, and HSP18.2) but also ASCORBATE PEROXIDASE 2. Their expression pattern is in strong contrast to that of HS-inducible non-memory genes such as *HSP70* and *HSP101*, whose expression peaks soon after HS and declines relatively quickly ^19, 29^. *HSFA2* was reported to be required for the maintenance of high expression levels of several HS memory-related genes but not for their induction, suggesting that they could be direct targets of HSFA2 ^17, 28^. Indeed, HSFA2 associates with the promoter of several of these genes *in vivo,* as demonstrated by chromatin immunoprecipitation ^29^. Interestingly, binding of HSFA2 to its target loci was detected only transiently, whereas active transcription was detected for much longer ^29^. Among the HS memory-associated genes is *HSA32*, which was the first gene that was specifically implicated in HS memory ^18^. Although *HSA32* has no homology to chaperones, it was reported to be required for HSP101 protein stability and thus may have a similar function ^30^. The peptidyl-prolyl-isomerase (and member of the FK506-binding protein family) ROF1 is also specifically required for HS memory ^31^. ROF1 was shown to directly interact with HSP90.1 and through HSP90.1 with HSFA2 ^31^. In *rof1* mutants, sustained induction of several target genes of HSFA2 was compromised, suggesting that ROF1 (together with HSP90.1) may maintain HSFA2 in an active state during the memory phase ^31^.

## Transcriptional memory of heat stress

As described above, both memory genes and non-memory heat-inducible genes are induced by HS, but only the former maintain very high expression levels for several days. To start to address the question of how these genes maintain such high and sustained expression levels, Lämke *et al.* investigated histone modification patterns at these loci during the memory phase ^29^. Using chromatin immunoprecipitation with histone modification-specific antibodies, the authors found that sustained induction of these memory genes was associated with sustained accumulation of histone H3 lysine 4 trimethylation and dimethylation (H3K4me3 and H3K4me2) that persisted even after active transcription from the loci had subsided. This raises the intriguing possibility that H3K4 methylation marks a locus as recently active and mediates a modified re-induction profile upon a second HS. Indeed, the memory gene with the highest accumulation of H3K4me3 and H3K4me2 showed a pronounced hyper-induction upon recurring HS ^29^. Notably, this H3K4 methylation is dependent on functional HSFA2 and is independent of the initial HS-mediated induction of the locus (which is also found in *hsfa2* mutants). As mentioned above, HSFA2 associates only transiently with HS memory loci during the early hours after HS, suggesting that it recruits other factors that mediate lasting chromatin modifications ^29^. Interestingly, HSFA2 itself appears not to be required for the maintenance of those chromatin changes ^29^. Thus, it will be revealing to learn more about the mode of action of HSFA2 in the future. Notably, H3K4 methylation has also been implicated in the memory of other abiotic stresses such as drought and salinity ^8, 9^. How H3K4 methylation is recruited in those cases and whether there is a common mechanism remain challenges for future studies.

## Heat stress memory at the protein level

Although regulation at the transcriptional level plays an important role in HS memory as described above, regulation at other levels may contribute to the memory. One such level may be regulated protein stability. For *HSP101*, transcript levels decline strongly within 24 hours after a priming HS; however, protein levels remain high for at least 48 hours ^29, 30^. During HS memory, HSP101 acts in a positive feedback loop together with HSA32, in which both proteins stabilize each other ^30^. This suggests that HSA32, whose function is still poorly understood, acts to prevent denaturation and degradation of proteins. This specific function of HSP101 during HS memory could be uncovered through the isolation of a specific missense mutation in the protein (T599I) from a genetic screen ^30^. The mutant HSP101 (T599I) protein was able to complement a yeast HSP104 deletion mutant, suggesting that its chaperone activity is not affected. In *A. thaliana*, this mutation specifically disrupts the function of HSP101 during HS memory but not during basal thermotolerance or during acquisition of thermotolerance. This suggests that the conserved chaperone activity of HSP101 is dispensable during HS memory. In rice, HSP101 and HSA32 stabilize each other in a similar manner ^32^. It is tempting to speculate that genes whose transcription depends on HSFA2 will be regulated at the level of transcription but that genes whose transcription is independent of HSFA2 will display high protein stability.

## Integrating stress exposure and development

As described above, an immediate effect of HS priming is to protect the plant during a recurring stress event. A more indirect effect may be the re-adjustment of growth and development after stress exposure. How this may be achieved molecularly became apparent through the finding that a microRNA (miRNA) family, which is important for plant development, is also required for HS memory ^19^. MiRNAs are short RNAs that associate with effector proteins to promote cleavage of complementary mRNAs or to inhibit their translation ^33^. The authors identified miRNAs whose expression is upregulated after HS and identified among them several *MIR156* isoforms. Overexpression of *MIR156* boosted HS memory, and depletion of *miR156* compromised it. In addition, *ARGONAUTE1* ( *AGO1*) was specifically required for HS memory but not HS priming. Several target genes of *miR156* whose transcript levels are reduced after HS were identified. *SQUAMOSA PROMOTER BINDING PROTEIN-LIKE* ( *SPL*) genes are well-studied target genes of *miR156* ^34^, and *SPL2*, *SPL9*, and *SPL11* were identified as relevant in the context of HS memory ^19^. They are downregulated after HS and this was dependent on a functional AGO1 protein and on the miRNA-binding site ^19^. Expression of a *miR156*-resistant form of *SPL2* and *SPL11* compromised HS memory, indicating that the repression of *SPL2* and *SPL11* by HS is required for HS memory. *SPL* genes regulate several aspects of development such as leaf initiation rate and flowering time ^34^. To separate the two functions, *miR156* levels were manipulated specifically after HS by using a heat-inducible promoter, and it was shown that the developmental effects are independent of the function during HS memory. Taken together, the *AGO1-miR156-SPL* module is important for plant development and also for HS memory. Although direct proof is yet elusive, it is tempting to speculate that employing the same miRNA/transcription factor module in stress acclimation and development may be used to integrate development with stressful environmental conditions ^10^.

## The evolution of heat stress memory

Given that plants acquire thermotolerance within minutes to hours after the onset of HS, the question remains as to why HS memory provides an adaptive advantage over *de novo* acclimation. To address this question, experiments with wild-type and memory-deficient genotypes under natural conditions will be required. Alternatively, natural and breeding-induced variation could be exploited to address this topic. A first step in this direction was undertaken by Charng and colleagues, who compared heat responses in two rice subspecies: the *Oryza sativa* ssp. *japonica* variety Nipponbare and the *O. sativa* ssp. *indica* variety N22 ^32^. *Indica* cultivars are thought to be more adapted to subtropical climates, whereas *japonica* cultivars grow in temperate climates ^32^. Interestingly, Nipponbare has a lower basal thermotolerance but higher HS memory capacity, whereas the *indica* variety N22 had a higher basal thermotolerance and a lower HS memory. It is tempting to speculate that a memory of past HS may be beneficial especially in temperate climates, where HS is a relatively rare event, compared with subtropical climates, where HS is frequent. Further studies will be needed to test this idea.

## Conclusions

Temperature stress is highly fluctuating in nature. Consequently, the priming and memory of HS may be beneficial for plant survival and fitness under natural environments. HS priming and HS memory in *A. thaliana* and rice have been established as model systems in which to study the molecular basis and evolution of priming and memory in response to abiotic stress. Although exciting progress has been made in recent years, we are still far from a mechanistic understanding. However, the emerging picture is that HS memory is regulated at different levels ranging from protein stability to miRNA-controlled mRNA stability to transcriptional memory. A challenge for the future will be to unravel how these different levels of control are integrated to achieve a robust physiological response. The ultimate goal of these studies is to mine the mechanistic knowledge gained in model organisms to unlock new approaches for breeding more heat-tolerant crop plants.

## Abbreviations

AGO1, ARGONAUTE1; HS, heat stress; HSF, heat shock transcription factor; HSP, heat shock protein; H3K4me, histone H3 lysine 4 methylation; miRNA, microRNA; SPL, SQUAMOSA PROMOTER BINDING PROTEIN-LIKE (SPL)
